# Scavenger Receptor C1 Mediates Toxicity of Binary Toxin from *Lysinibacillus sphaericus* to Ag55 Cells

**DOI:** 10.3390/toxins16080369

**Published:** 2024-08-21

**Authors:** Qi Zhang, Gang Hua, Laramie Smith, Michael J. Adang

**Affiliations:** 1 College of Plant Protection, Shenyang Agricultural University, Shenyang 110866, China; qizhang@syau.edu.cn; 2 Department of Entomology, University of Georgia, Athens, GA 30602-2603, USA; 3 Department of Biochemistry and Molecular Biology, University of Georgia, Athens, GA 30602-2603, USA

**Keywords:** *Lysinibacillus sphaericus*, Bin toxin, scavenger receptor C1, RNA interference, Ag55 cells, endocytosis

## Abstract

*Lysinibacillus sphaericus* harboring Binary (BinA and BinB) toxins is highly toxic against *Anopheles* and *Culex* mosquito larvae. The *Anopheles* Ag55 cell line is a suitable model for investigating the mode of Bin toxin action. Based on the low-levels of α-glycosidase Agm3 mRNA in Ag55 cells and the absence of detectable Agm3 proteins, we hypothesized that a scavenger receptor could be mediating Bin cytotoxicity. Preliminary RNA interference knockdown of the expressed scavenger receptors, combined with Bin cytotoxicity assays, was conducted. The scavenger Receptor C1 (SCRC1) became the focus of this study, as a putative receptor for Bin toxins in Ag55 cells, and SCRBQ2 was selected as a negative control. Open reading frames encoding SCRC1 and SCRBQ2 were cloned and expressed in vitro, and polyclonal antibodies were prepared for immunological analyses. The RNAi silencing of SCRC1 and SCRBQ2 resulted in the successful knockdown of both SCRC1 and SCRBQ2 transcripts and protein levels. The cytolytic toxicity of Bin against Ag55 cells was severely reduced after the SCRC1-RNAi treatment. The phagocytic receptor protein SCRC1 mediates endocytosis of the Bin toxin into Ag55 cells, thereby facilitating its internal cytological activity. The results support a mechanism of the Bin toxin entering Ag55 cells, possibly via SCRC1-mediated endocytosis, and encourage investigations into how Bin is transferred from its bound form on the midgut epithelial cells into the epithelial endocytic system.

## 1. Introduction

Mosquitoes are primary vectors that transmit infectious pathogens to humans and other mammals [1]. Female mosquitoes of some species ingest pathological microorganisms from infected human or animal hosts during a blood meal, with the ingested pathogens replicating in mosquitoes, which is then followed by transmission to a new host during the next blood meal [2]. Three major pathogen-transmitting mosquito genera (*Anopheles*, *Culex*, and *Aedes*) are responsible for spreading parasites and viral diseases such as malaria, dengue, yellow fever, and Japanese encephalitis, which combined cause more than 700,000 deaths yearly worldwide (https://www.who.int/news-room/fact-sheets/detail/vector-borne-diseases (accessed on 4 April 2019).

Mosquito larvicides based on insect pathogenic bacteria are important tools in mosquito management. *Bacillus thuringiensis* variety *israelensis* is widely used to control the larval stages of pathogen-transmitting mosquitoes [3]. A second bacterium, *Lysinibacillus sphaericus*, is also an important larvicide, especially due to its efficacy against the *Culex* species and its good activity against *Anopheles* species.

The insecticidal efficacy of *L. sphaericus* is due primarily to its Binary (Bin) toxin proteins, which are deposited in a parasporal crystal. The Bin toxin is composed of the 42 kDa BinA and 51 kDa BinB proteins [4]. The Bin A and B subunits work together to confer toxicity, as BinB serves as a receptor binding component, while Bin A is responsible for toxicity inside the target cells. This general model for Bin A and B action is consistent between mosquito larvae [5] and cancer cells [6]. BinA plus BinB together exhibit high toxicity against *Culex* and *Anopheles* larvae [5]. In susceptible *Culex* larvae, the ingested and solubilized BinAB complex binds an α-glucosidase tethered to the midgut cells [7]. Bin-resistant *Culex* larvae lack the target α-glucosidase and are not killed by *L. sphaericus* and Bin toxin. Bin also binds an α-glucosidase (Agm3) in *A. gambiae*, which presumably functions as a receptor protein. The Bin toxin acts intracellularly in *Culex* larvae, becoming localized within the endocytic apparatus and mitochondria [8]. *Culex* larvae intoxicated by Bin show evidence of autophagy and apoptosis in the midgut tissue [5,9].

Ag55 cells, recently determined to originate from *Anopheles coluzzii* [10], are competent for the processing of exogenous full-length double-stranded RNA (dsRNA), allowing for the silencing of genes by RNAi [11]. Importantly, Ag55 cells are a means for investigating Bin toxin action [12,13]. While Bin recognizes Agm3 α-glucosidase in larvae, evidence suggests that Agm3 does not function as a receptor for Bin in Ag55 cells. Nevertheless, Bin is toxic to Ag55 cells, and the toxin enters the cells via their endocytosis system, induces vacuolation, and localizes to the lysosomes before cell lysis [12]. Bin rapidly decreases mitochondrial respiration and ATP production in Ag55 cells [13]. Using Ag55 cell proteins and a proteomics-based approach, Riaz et al. [13] identified clathrin, an endocytosis protein, and several glycolytic enzymes as Bin-interacting proteins. How the Bin toxin enters Ag55 cells is the subject of this study.

Cultured *Drosophila* S2 and Ag55 cells are phagocytic and have phagocytic receptors that bind to molecules on the surface of pathogens and apoptotic cells [10,14]. The transcriptomic analysis of Ag55 cells identified the expression of sixteen putative scavenger receptors, based on their homology to known *Drosophila* scavenger receptor proteins [10]. Among the scavenger receptor families, proteins in Class C were only identified in insects and were not found in mammalian organisms [15,16,17]. The scavenger receptor C has conserved functions regarding host defense responses in *D. melanogaster* [15]. Further analysis of the scavenger family members was conducted in the aspects of functional domains and conserved amino acid sequences of families A to D in *D. melanogaster* and *D. simulans* [16]. Except for family C, the scavenger B family protein was also determined in *Drosophila* to work as a cholesterol carrier [18]. Considering its potential role in insect pathogenic bacteria, the scavenger receptor C was first reported to work as a functional receptor, on Sf9 cells, for the *B. thuringiensis* Vip3Aa toxin, binding to the Vip3Aa toxin with a high affinity [19].

The overall objective of this study is to investigate the scavenger receptors expressed in Ag55 cells as possible receptors for the Bin toxin. While the targeting of scavenger receptors in this study is contrary to the Bin-interacting proteins we reported in [13], the investigations overlapped. Based on the results from a small-scale preliminary screening of Bin’s toxicity to Ag55 cells, having knocked-down levels of scavenger receptors by RNAi, attention turned to SCRC1 as a putative receptor for Bin toxicity in Ag55 cells. Our results showed that the cytotoxic effects of the Binary toxin against Ag55 cells were nearly eliminated when the scavenger receptor C was silenced, providing evidence for a functional role in regulating Binary toxin action in Ag55 cells.

## 2. Results

### 2.1. Analysis of SCRC1 and SCRBQ2 Predicted Proteins

The analysis determined that the Ag55 SCRC1 cDNA is identical to the predicted SCRC1 ORF of *A. arabiensis* (XP_040235850.2, Figure S1). Similarly, the cloned *SCRBQ2* cDNA is nearly identical to the predicted SCRBQ2 protein (XP_040167406.1, Figure S2) of *A. arabiensis*, with a difference of only one amino acid. The ORFs of both these proteins and their related annotations are available by their XP numbers in VectorBase (https://vectorbase.org/vectorbase/app (accessed on 21 November 2018). The *SCRC1* ORF is 2699 bp, encoding 752 amino acids (Figure S1), and yielding an estimated 120 kDa SCRC1 protein; the length of *SCRBQ2* is 1681 bp, encoding 492 amino acids, and resulting in an 80 kDa protein (Figure S2). The SCRC1 and SCRBQ2 proteins have a signal leading peptide and a transmembrane domain, as indicated in Figures S1 and S2. The SCRC1 protein, but not SCRBQ2, has a C-terminal domain that is rich in threonines. SCRC1 contains a Complement Control Protein (CCP) domain (aa 49–103) and a MAM domain (aa 162–335). These are evolutionarily conserved protein domains that exist in a wide variety of complement-type proteins with adhesive functions. There are no predicted CCP and MAM domains present in the SCRBQ2 protein; however, the SCRBQ2 has a predicted CD36 domain, which is putatively involved in immunity and metabolism.

### 2.2. RNAi-Based Reduction in SCRC1 and SCRBQ2 Transcript Levels in Ag55 Cells

The RNAi experiments with dsRNAs and Ag55 cells were performed with two SCR-dsRNAs combined in a treatment. The transcript levels of SCRC1 and SCRBQ2 in the cells were measured by RT-PCR 48 h after the onset of the dsRNA treatments. The Ag55 cells were incubated with the eGFP-dsRNA alone as a control and with dsSCRC1.

**Figure 1:**
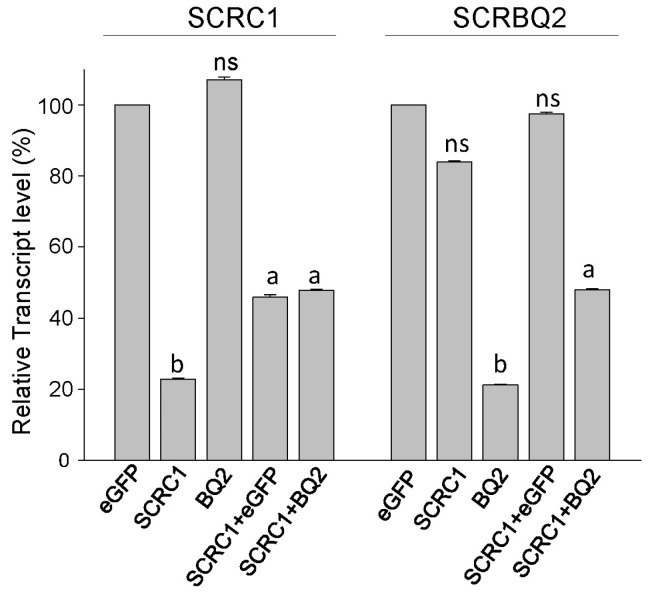
Knockdown of SCRC1 and SCRBQ2 expression in Ag55 cells. The dsRNAs, dseGFP, dsSCRC1 and dsSCRBQ2 were synthesized as described in Section 4. Relative amount of SCRC1 and SCRBQ2 transcripts in each treatment group was compared to that of eGFP-dsRNA after normalization to the expression of AgRPS3. Statistically different values are designated by different letters, whereas values statistically the same as eGFP controls are designated ns.

**Figure 2:**
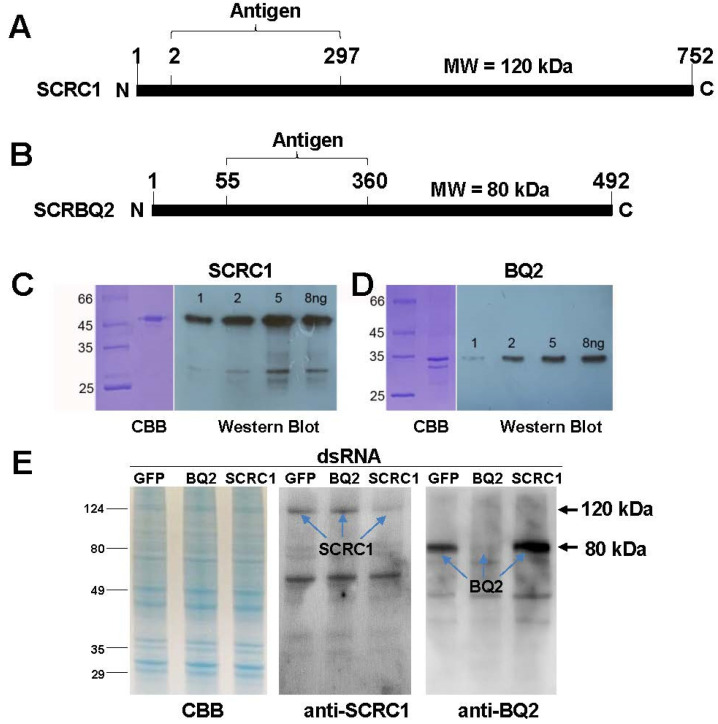
Western blot analysis of SCRC1 and SCRBQ2 produced in *E. coli* and in Ag55 cells after RNAi treatments. (**A**,**B**) Diagrams of SCRC1 and SCRBQ2 proteins and regions expressed for use as antigens. (**C**,**D**) Partially purified SCRC1 and SCRBQ2 proteins produced in *E. coli*, stained with Coomassie brilliant blue (CBB), and detected by Western blot as described in Section 4. (**E**) SCRC1 and SCRBQ2 proteins in Ag55 cells stained with CBB and detected on Western blots after dsRNA interference as described in Section 4.

The levels of the *SCRC1* and *SCRBQ2* transcripts were reduced about 80% by the dsRNA treatments with each respective full-length dsRNA (Figure 1); no cross-interference was detected between the SCR dsRNAs. The negative control of eGFP-dsRNA did not alter the SCRC1 or SCRBQ2 transcript levels. As shown in Figure 2, an unexpected reduction in the inhibition of SCR transcripts, referred to as a ‘buffering effect’, was observed when the dsSCRC1 and dsSCRBQ2 RNAs were combined in the treatments of the Ag55 cells. After the dsRNA treatments, the SCRC1 and SCRBQ2 transcript levels were reduced to only 45% of the non-treated level, rather than to the expected treatment expression level of 20% (Figure 1). However, the buffering effect did not occur when eGFP was the second dsRNA. Collectively, the dsSCR RNAs effectively and specifically reduced the targeted SCR transcripts to about 20% of the control levels.

### 2.3. Anti-SCRC1 and Anti-SCRBQ2 Sera Detection of SCRC1 and SCRBQ2 Proteins

Anti-SCRC1 and SCRBQ2 sera were used to evaluate the efficacy of the dsSCR RNAi experiments in the Ag55 cells by protein blot analysis. Figure 2A,B show diagrams of the SCRC1 and SCRBQ2 proteins and the regions that were expressed in *E. coli* for rabbit antiserum production. Each antiserum can detect 1 ng of its respective *E. coli*-produced antigen at a 1:1000 dilution (Figure 2C,D). The signals with the anti-SCRC1 serum were visibly stronger than with the anti-SCRBQ2 serum for the same amount of the target protein (Figure 2C,D).

For the dsRNAi experiments, the Ag55 cells were treated with the SCRC1 or SCRBQ2 dsRNA for three days and then the cells were harvested and disrupted in an SDS sample buffer. The proteins were separated by SDS-PAGE, transferred to a PVDF filter, and probed with the anti-SCRC1 or anti-SCRBQ2 serum. As shown in Figure 2E, the dsSCRC1 treatment of the Ag55 cells visibly reduced the signal for SCRC1 detected by the anti-SCRC1 serum. Similarly, the band corresponding to the SCRBQ2 protein was reduced to a nearly non-detectable level after the dsSCRBQ2-RNA treatment of the Ag55 cells. As visible in the anti-SCRC1 and anti-SCRBQ2 blot panels, the control ds-GFP and dsSCR treatments showed no reductions in their SCR band intensity (Figure 2E). These results support the transcript-level results, demonstrating that the dsRNA-SCRC1 and dsRNA-SCRBQ2 not only specifically reduced the transcript levels, they also specifically reduced the levels of the targeted SCR proteins. 

### 2.4. Bin Toxicity to Ag55 Cells Treated with dsRNAs

**Figure 3:**
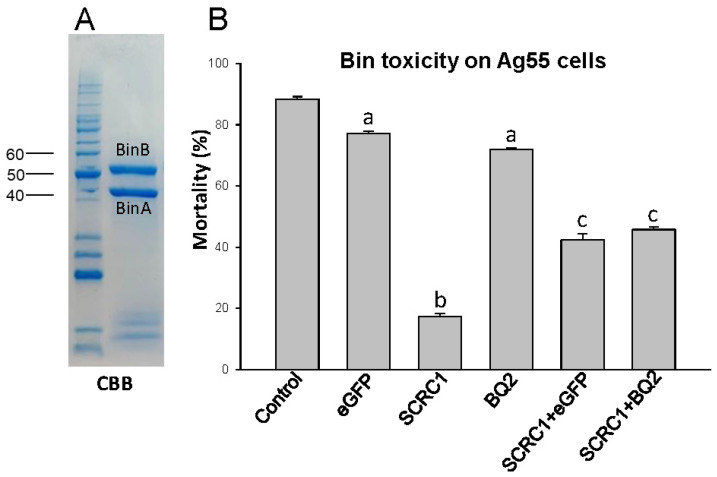
Toxicity of Bin toxin on Ag55 cells treated with dsRNAs. After dsRNA treatments for three days, Ag55 cells from the control (buffer or eGFPdsRNA) and experimental groups (SCRC1-dsRNA and SCRBQ2-dsRNA) were bioassayed. (**A**) Coomassie brilliant blue (CBB) stained BinA and BinB subunits of Binary toxin. (**B**) Bioassays were performed with 25 nM of Bin toxin and cell mortality was recorded on day 2. Each data point represents the mean ± standard error of the results from bioassay. A significant difference (chi-square analysis; *p* < 0.001) was obtained between cell mortality with ds-eGFP and ds-SCRC1 (a vs. b). The significances (*p* < 0.001) are also presented between ds-eGFP (or ds-SCRBQ2) and ds-SCRC1 + eGFP (or ds-eGFP and ds-SCRC1 + BQ2) (a vs. c).

The Ag55 cells were assessed for their susceptibility to the Bin toxin after treatments with the SCR dsRNAs. The BinA and BinB components, resulting from solubilization and trypsin activation of Bin crystals, are shown as stained bands on the gel in Figure 3A. The Ag55 cultured cells were treated with the dsRNA, and then incubated with 25 nM of the activated Bin toxin for 48 h. Cell mortality was assessed based on trypan blue exclusion (Figure 3B). Only treatments containing the dsSCRC1 protected the Ag55 cells from the Bin toxin. After the SCRC1-dsRNA treatment, the Bin-induced mortality decreased to 17.4 ± 1.1% (*p* < 0.001), as compared to 87.4 ± 1.0% for the control (no dsRNA) cells. The Bin-induced mortality, at 72% for the SCRBQ2-dsRNA treated cells and 78% mortality for the GFP-dsRNA treated cells, was not significantly different from the control untreated cells (*p* > 0.05) (Figure 3B). It is notable that the Bin toxicity to the Ag55 cells treated with dsSCRC1 RNA was buffered, that is, the Bin protection effect was partially neutralized when cells were treated with the combination of SCRC1/SCRBQ2-dsRNA (45.0 ± 0.8%) or SCRC1-/eGFP-dsRNA (42.4 ± 1.5%), as compared to treatments with the SCRC1-dsRNA alone (17.4 ± 1.1%) (Figure 3B). The Ag55 cells treated with the activated Bin toxins became round and vacuoles appeared in the cytoplasm, which was followed by cell degradation. The Ag55 cells that were protected from Bin by the knockdown of SCRC1 appeared the same as the untreated cells at the light microscope level.

**Figure 4:**
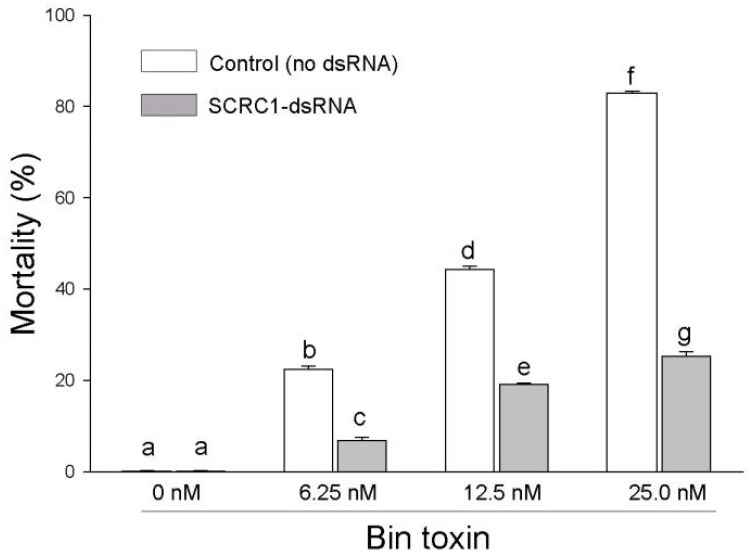
Bin toxicity to Ag55 cells treated with or without SCRC1-dsRNA. Ag55 cells were exposed to Bin toxin at a final concentration of 0, 6.25, 12.5, or 25 nM for 48 h and cell mortalities were measured by trypan blue exclusion. Cell mortality is expressed as the % dead cells relative to total cells without Bin treatment. Error bars show standard error of the means. Letters a–g denote significant differences between compared values (*p* < 0.05).

The possible effects of the Bin treatment concentrations on the Ag55 cells and the dsRNA-based inhibition of SCRC1 were measured for the control and dsSCRC1-treated cells at three Bin concentrations. Cell mortality increased with increasing Bin concentrations from about 20% to 45% to 80%; pretreatment with the ds-SCRC1 RNA protected the Ag55 cells with a (*p* < 0.001) at each Bin concentration (Figure 4).

## 3. Discussion

Published studies from our team have shown that *L. sphaericus* Bin toxin kills Ag55 cells [12,13]. However, α-glucosidase (Agm3), the putative receptor for Bin in *A. gambiae* larvae, has a very low transcript level in Ag55 cells [10] and is below a detectable level by Western blot analyses (G. Hua, personal communication). Consequently, Agm3 α-glucosidase most likely does not function as a Bin receptor in Ag55 cells. Herein we show that the scavenger receptor SCRC1 mediates Bin cytotoxicity to Ag55 cells.

The cDNAs encoding Ag55 SCRC1 and SCRBQ2 were used to design PCR primers (Table 1) and produce SCRC1 and SCRBQ2 proteins in *E. coli*. Unexpectedly, the encoded proteins were identical to the *A. arabiensis* proteins (XP_040167338.1) and (XP_061510019.1), respectively. Ag55 SCRC1 differs from *A. coluzzii* (XP_040235850.2) by six amino acids internally within the predicted protein. The absence of the predicted eleven N-terminal residues from the *A. coluzzii* SCRC1 protein could be a consequence of our primer design being based on the *A. gambiae* SCCR1 protein, which lacks those putative N-terminal amino acid residues. SCRBQ2 differs from *A. coluzzii* by two amino acids. Alignments for the Ag55 SCRC1 and SCRBQ2 against homologues in *A. gambiae*, *A. arabiensis*, and *A. coluzzii* are presented in Figures S1 and S2. Considering that *Aedes aegyptii* is not susceptible to *L. sphaericus* Bin toxin, we searched genome and scientific literature databases for homologues to AgSCRC1 (AGAP011974). We identified XP_021710624, with 39.8% identity, and AAEL006361 [20], with 34.0% identity. Figure S3 has a description of how these homologues were identified and a ClustalW alignment of the two *Aedes* SCRC proteins against AgSCRC1.

We effectively decreased the mRNA levels of SCRC1 and SCRBQ2, by applying dsRNA to the medium of Ag55 cell cultures, to about 80% below the control levels as measured by RT-PCR (Figure 1). The efficacy of dsRNA in these RNAi experiments supports the observation of an effective RNAi system in Ag55 cells by Smith and Linser [11]. Ulvila et al. [21] reported that SCRCI and Eater together led to more than a 90% decrease in the internalization of dsRNA and uptake into the S2 cells when compared with the GFP dsRNA treated controls [21]. If SCRC1 does internalize dsRNA into Ag55 cells, as we suspect, it could account for the ‘buffering’ effect that we refer to. For example, if dsRNA is taken into cells via SCRC1, then a decreased amount of SCRC1 on the cell membrane would reduce the capacity of the Ag55 cells to take in dsRNA. It is possible that when dsSCRC1 is combined with a second dsRNA, such as dsSCRBQ2 in a treatment, the amount of dsRNAs is greater than the uptake capacity of Ag55 cells. The result is what we call a ‘buffering effect’. The observed decrease in SCRC1, and possibly decreased endocytosis, would result in a decrease in dsRNAs internalizing into cells. The decreased SCRC1 and SCRBQ2 transcript levels by RNAi, was buttressed at the protein level by Western blot analyses with the specific anti-SCR sera (Figure 2). Importantly, specific knockdown of the SCRC1 transcript and protein levels directly correlated with the reduced Bin toxicity to the Ag55 cells (Figure 3 and Figure 4). These results support the role of SCRC1 as a functional receptor for Bin in Ag55 cells.

This is the first report of the functional effect of SCRC1 as a Bin toxin regulator and is in accordance with a recent study on the Vip toxin, which shows that the scavenger C protein works as a putative receptor for Vip3Aa toxin with a high binding affinity on *S. frugiperda* Sf-9 cells [19]. However, we have not presented direct evidence that SCRC1 binds Bin and internalizes the toxin through endocytosis. The scavenger receptor SCRC1 is, to our knowledge, found only in insects including fruit flies and mosquitoes; SCRC1 also participates in the innate immune response against bacterial pathogens [15,16,17].

The reduction in SCRC1 mediated by dsRNA-SCRC1 at the transcript level was mirrored at the protein level and corresponded to the reduced Bin cytotoxicity. The residual SCRC1 protein remaining after RNAi was visible in Western blot analysis (Figure 2) and could account for the remaining 20% of Bin-induced toxicity to Ag55 cells. An anomaly in the RNAi results is that the treatment of cells with the combination of dsRNA-SCRC1 and dsRNA-SCRBQ2, but not dsRNA-SCRC1 plus dsRNA-eGFP, reduced the efficiency of RNAi at the transcript level. Yet, both combinations of dsRNAs reduced the cytotoxicity of Bin to Ag55 cells (Figure 3). Due to this discrepancy, we cannot say the uptake of dsRNAs exceeded a maximal uptake level, though the RNAi of SCRC1 could impact dsRNA uptake. Two *Drosophila* scavenger receptors, SCRC1 and Eater, account for more than 90% of dsRNA uptake into Drosophila S2 cells [10,14]. It should be noted that transcripts of Eater were not detected in Ag55 cells [10].

The function of SCRC1 as a mediator of Bin toxicity to Ag55 cells could add insights into a previous observation that pitstop2, an inhibitor of clathrin-mediated endocytosis, synergizes Bin toxicity to Ag55 cells [13]. Clathrin is involved in cell phagocytosis, which delivers large particles into lysosomes for degradation [22]. It is possible that SCRC1 mediates Bin entrance, which then results in Bin tracking into lysosomes and phagolysosomes. Possibly, some Bin also enters cells via clathrin-mediated endocytosis and that mechanism does not result in cell death. If the clathrin route is inhibited by pitstop2, then SCRC1-mediated access to the endosomal system could be enhanced, leading to increased cell death due to Bin intoxication.

This is the first report of SCRC1 from *Anopheles* functioning as a regulator of Bin toxin on Ag55 cells. It is important to determine if there are direct interactions between Bin and SCRC1. Additionally, it will be interesting to investigate if SCRC1 also is involved in Bin toxicity to mosquito larva by co-operating with Bin’s receptor, α-glucosidase. Further investigation concerning how SCRC1 mediates Bin toxin in the cytolytic process to exert apoptosis or autophagy to Ag55 cells is highly warranted.

## 4. Materials and Methods

### 4.1. Ag55 Cells

The Ag55 cell line originated from *A. gambiae* strain M [23], which was reclassified as *A. coluzzii* while remaining in the *A. gambiae* complex [24]. Ag55 cells were recently confirmed to be of *A. coluzzii* origin [10]. Stocks of this cell line have been maintained in liquid nitrogen in the Adang lab for over 12 years and a fresh vial of cells from stocks was ‘recovered’ for this research. Ag55 cells were cultured as previously described [12]. Ag55 cells showing >70% confluence in 25 cm^2^ flasks were used for dsRNA treatments. Briefly, 3 × 10^6^ cells/well were seeded in 6-well plates (Corning, Corning, NY, USA) with 2 mL of fresh medium and treated with 20 µg of dsRNA for three days. The dsRNA-treated cells were divided into two parts, of which one was used for total RNA extraction and the other for bioassay.

### 4.2. L. sphaericus and Bin Protein Preparation

*L. sphaericus* strain ISPC-8 was cultured for experimentation as reported by [12]. Cultures were harvested when most (>90%) of the bacteria in culture had sporulated, as observed by phase contrast microscopy. Crystals in the washed spore/crystal preparation were solubilized in 50 mM NaOH on ice for 3 h and the resultant soluble Bin protoxin was activated with trypsin (Sigma, Darmstadt, Germany) in a mass ratio of 20:1 (protein/trypsin) at 37 °C for 2 h. Activated Bin was purified with a HighQ cartridge (BioRad, Hercules, CA, USA) according to Hire et al. [12]. The activated BinAB protein mixture was separated by 12% SDS-PAGE, stained with Coomassie Blue, visualized using an imaging station (AlphaInnotech, San Leandro, CA, USA) and quantified by BioRad protein assay using BSA as a standard. Purified Bin toxin was aliquoted and kept in −80 °C for cell culture toxicity assays.

### 4.3. Synthesis of Partial SCRC1 and SCRQB2 Regions for dsRNA Experiments

The cDNA from Ag55 cell mRNAs was synthesized with reverse-transcriptase III (Invitrogen, Carlsbad, CA, USA) using total RNA extracted by TriZol reagent (Sigma, Darmstadt, Germany). The resultant cDNA was used to amplify Class C Scavenger Receptor (SCRC1) (VectorBase: AGAP011974) and Class B Scavenger Receptor (SCRBQ2) (VectorBase: AGAP010133) partial regions by PCR. We performed 1st PCR and nested PCR with primers (Table 1: Ag1–4) to obtain an SCRC1 partial gene, which was used as template for another PCR with region-specific primers (dsRNA-SCRC1-f and -r), tailed with the T7 polymerase promoter sequence. The primers of Ag5–8 and dsRNA-SCRBQ2 (Table 1) were used to PCR-amplify SCRBQ2 partial gene tailed with T7 promoter. Amplification was performed using 2 × Taq Master Mix (ProbeGene, Xuzhou, China). PCR reaction conditions were performed as follows: 5 min at 94 °C, 30 cycles of 30 s at 94 °C, 30 s at 55 °C, and 60 s at 72 °C. After the amplicons were confirmed by sequencing, SCRC1-dsRNA or SCRBQ2-dsRNA were synthesized in vitro with the Ambion MEGA-script high yield transcription kit (Applied Biosystems/Ambion, Austin, TX, USA) according to the manufacturer’s protocols. A control dsRNA (eGFP-dsRNA) was also synthesized in the same way using primers dsRNA-eGFP-f and dsRNA-eGFP-r (Table 1). Purified dsRNAs were quantitatively determined by NanoDrop (N-1000) spectrophotometer and stored at −20 °C for treatment on Ag55 cells.

### 4.4. RNAi Silencing SCRC1 and SCRBQ2 Expression in Ag55 Cells and RT-qPCR Quantitation

Freshly cultured Ag55 cells in flasks (Corning) were treated with 20 µg dsRNA in 10 mL medium (Sigma) for three days. Three days later, the cells were evaluated for transcript levels by quantitative real-time PCR (RT-qPCR) with the primers (Table 1). Three groups of cells from each treatment were separately soaked in 200 μL of TRIzol reagent (Ambion, Austin, TX, USA) in a microfuge tube, homogenized with a cordless motor-driven pellet pestle (Grainger, Chicago, IL, USA), and centrifuged at 12,000× *g* for 30 min at 4 °C. The supernatant was collected and mixed by shaking vigorously with 40 μL of chloroform. The mixture was set at room temperature for 5 min and centrifuged as above for 15 min at 4 °C. The upper aqueous phase, containing RNA, was collected into a new tube and mixed with the same volume of 100% isopropanol. After incubation at room temperature for 10 min, the mixture was centrifuged at 12,000× *g* for 10 min at 4 °C. The RNA pellet was washed with 75% ethanol, air dried, and dissolved in 50 μL of RNase-free water. The total RNA amount was determined with NanoDrop Spectrophotometer (N-1000).

The cDNA was synthesized with SuperScript III First-Strand Synthesis System (Invitrogen) using 5 μg of total RNA as template. The resulting cDNA was diluted 100-fold for RT-qPCR to value gene transcripts. We used iQ SYBR Green Supermix (Bio-Rad) primers added as follows: SCRC1/qPCR-f and SCRC1/qPCR-r for *SCRC1* expression; SCRBQ2/qPCR-f and SCRBQ2/qPCR-r for *SCRBQ2* expression; and AgRPS3/qPCR-f and AgRPS3/qPCR-r (Table 1) for the endogenous control (RPS3), which were selected based on 40S ribosomal protein S3 (AGAP001910-RA). Relative percentages of gene silencing were calculated from three biological replicates of each treatment using RPS3 to normalize gene expression by the 2^−ΔΔCT^ method.

### 4.5. Construction of SCRC1 and SCRBQ2 Coding Regions in Plasmid Expression Vectors

The sequences of SCRC1 (VectorBase: AGAP011974) and SCRBQ2 (VectorBase: AGAP010133) genes were analyzed based on the genomic database of *A. gambiae* in VectorBase database. Plasmids harboring SCRC1 and SCRBQ2 genes were constructed using NdeI-XhoI enzymatic sites to express these two genes in *E. coli*. Amplification was performed using 2 × Taq Master Mix (ProbeGene). PCR reaction conditions were as follows: 5 min at 94 °C, 30 cycles of 30 s at 94 °C, 30 s at 55 °C, SCRBQ2 60 s at 72 °C. 30 s at 55 °C, and 60 s at 72 °C. PCR products of SCRC1 and SCRBQ2 genes were cloned into pET-32T and pET-28a+ plasmids, respectively, yielding SCRC1-pET-32T and SCRBQ2-pET-28a constructs for protein expression. The DNA inserts were both sequenced by Sangon Biotech Company in both forward and reverse directions with the sequences identical to *A. gambiae* SCRC1 and SCRBQ2 respectively.

Plasmids of SCRC1-pET-32T and SCRBQ2-pET-28a were transformed into *E. coli* TOP10 competent cells and inoculated into fresh LB liquid medium (containing 0.01 mg/L kanamycin) at 37 °C for protein expression. The cultures were induced with 0.5 mM IPTG (Amersco, Framingham, MA, USA) when OD600 nm of the suspension reached 0.5–0.6 and cultured for another 15 h. The cells were collected and harvested for purification of expressed proteins. The cells were resuspended in buffer (20 mM Tris-HCl, 50 mM NaCl, 0.1% Triton X-100, pH 8.0) and lysed by ultrasound. The expressed SCRC1 or SCRBQ2 proteins were purified with Ni-IDA column (Biovision, Atlanta, GA, USA), eluted with 50 mL of buffer (20 mM Tris-HCl pH8.0, 2 M NaCl, 0.1% TritonX-100, and 20 mM imidazole). The purified proteins were checked using 12% SDS-Page electrophoresis and CBB staining.

### 4.6. Preparation of Polyclonal Antibodies against SCRC1 and SCRBQ2 Proteins

Inclusion bodies of SCRC1 and SCRSCRBQ2 were separately prepared and solubilized in buffer (50 mM Tris, 500 mM NaCl, 5 mM DTT, 8 M Urea, pH 8.0). Each supernatant containing soluble SCRC1 or SCRBQ2 proteins was collected after centrifugation at 15,000 rpm for 30 min; proteins were then re-natured (50 mM Tris, 50 mM NaCl, 500 mM L-Arginine, 3 mM GSSG, 1 mM GSH, 1 mM DTT, pH 8.0) at 4 °C for 48 h. Then, the protein samples were dialyzed using 20 mM Tris/50 mM NaCl pH 8.0, at 4 °C for 16 h. The final purity was 80% for SCRC1 and 70% for SCRBQ2 as assessed by ImageJ software (Version 1.52a). The purified proteins were used as antigens to immunize two New Zealand white rabbits (2–2.5 kg) with subcutaneous immunization at a volume of 400 µg/time for a total of 4 times. Blood samples were collected and the titer of each antiserum was determined against SCRC1 and SCRBQ2 by indirect ELISA and antisera was used for experimentation when the titer was greater than 1:50,000.

### 4.7. Western Blot Analyses

Purified SCRC1 and SCRBQ2 proteins (2 µg each) produced in *E. coli* were heated in SDS-PAGE sample buffer, separated by 12% SDS-PAGE, and then transferred to a PVDF filter. The filters were blocked (1× PBS, pH 7.4 + 5% skim milk) at room temperature for 1 h, then incubated with 1:1000 diluted antiserum in buffer (1× PBS-0.1% tween-20 + 0.1% skim milk) at room temperature for 3 h. After washing, filters were incubated with α-rabbit IgG–HRP (1:5000 dilution) in the same buffer for 1 h at room temperature. Final reacted bands were captured using GelDoc Go Imagine System (G:BOX, Chemi XX6, SYNGENE, Bangalore, India).

Ag55 cultured cells were treated with dsRNA for three days as described in Section 2.4. Whole cells (dsRNA-GFP, dsRNA-SCRC1, and dsRNA-CRBQ2 cells) were harvested by centrifugation at 400× *g* for 2 min followed by three washes with PBS. The same number of cells (1 × 106) were resuspended in SDS-PAGE sample buffer and centrifuged after boiling for 10 min. The supernatants were loaded on 12% SDS-PAGE (BioRad) for separating proteins. The separated proteins in the gel were transferred to a PVDF membrane (Millipore), which was in turn blocked in blocking buffer (1× PBS pH 7.4 + 5% skim milk) for 1 h. Then, the filters were probed with SCRC1 or QB2 antiserum (1:5000) in buffer (1× PBS-0.1% tween-20 + 0.1% skim milk) for 3 h. After washing, the filters were incubated with α-rabbit IgG–peroxidase conjugate (1: 25,000 dilution) in the same buffer for 1 h at room temperature. Final reacted bands were captured using GelDoc Go Imagine System (G:BOX, SYNGENE).

### 4.8. dsRNA Treatment and Cytotoxicity Assays

The fresh Ag55 cells in 25 cm^2^ flasks (Corning) were treated with 20 µg dsRNA in 10 mL medium (Sigma) for three days. The dsRNA-treated cells were divided into replicates in two wells of a 6-well plate for Bin cytotoxicity assays. Activated BinAB (25 nM each in 20 mM Na-phosphate buffer, pH 8.0) was applied to one well and the same volume of the buffer was added to the other well as a control. The mortality of Ag55 cells was assessed with trypan blue exclusion assay [12] 48 h later. To assess cytotoxicity of the quantities of BinAB toxin against dsRNA treated cells, activated BinAB was serially diluted in 100 μL of 20 mM Na-phosphate buffer (pH 8.0) and was added to cells cultured in 2 mL culture medium to achieve a final concentration of 0, 6.25, 12.5, or 25 nM of toxin. Two days later (48 h), the number of viable cells was counted as follows. Viable (excluding trypan blue) and non-viable (trypan stained) cells in 4 large squares (1 mm × 1 mm) of hemocytometer were counted and the average numbers of live cells were calculated. Cytotoxicity assays were independently repeated three times, and data from replicate samples were pooled for analysis and plotted using SigmaPlot (v. 11.0).

## Figures and Tables

**Table 1:** Nucleotide primers used in this study *.

Primers	Orientation	DNA Sequence
Ag1	Forward	5′-ATGCTGTTATCAAGAAGCTCG-3′
Ag2	Reverse	5′-CGGTCCCCTCAAAGCAGG-3′
Ag3	Forward	5′-GAAGCTCGAAGGGAGCAAC-3′
Ag4	Reverse	5′-CGATGTGTCCCAGCGACC-3′
Ag5	Forward	5′-GTTCACCATGTACAGACTTT-3′
Ag6	Reverse	5′-CGCCTTCGAACAGCAGCTC-3′
Ag7	Forward	5′-CACTAGGATGTTCGTCCTTTC-3′
Ag8	Reverse	5′-GCGCACTGGTTTGTTTTTCC-3′
dsRNA-SCRC1-f	Forward	5′-taatacgactcactatagggGAAGCTCGAAGGGAGCAAC-3′
dsRNA-SCRC1-r	Reverse	5′-taatacgactcactatagggCGATGTGTCCCAGCGACC-3′
dsRNA-BQ2-f	Forward	5′-taatacgactcactatagggCACTAGGATGTTCGTCCTTTC-3′
dsRNA-BQ2-r	Reverse	5′-taatacgactcactatagggGCGCACTGGTTTGTTTTTCC-3′
dsRNA-eGFP-f	Forward	5′-taatacgactcactatagggCACGACGTTGTAAAACGAC-3′
dsRNA-eGFP-r	Reverse	5′-taatacgactcactatagggGATAACAATTTCACACAGG-3′
SCRC1/qPCR-f	Forward	5′-GGTTTGTGTGAAATCCGGATG-3′
SCRC1/qPCR-r	Reverse	5′-CGGTCCCCTCAAAGCAGG-3′
BQ2/qPCR-f	Forward	5′-GCAGATCAATGAGCACTTGC-3′
BQ2/qPCR-r	Reverse	5′-CAGTGCAAGCTTCGCCTGT-3′
AgRPS3/qPCR-f	Forward	5′-TCCGCAAGCGTTTCGGATTC-3′
AgRPS3/qPCR-r	Reverse	5′-CACGGCCAGCCCACCGAT-3′
SCRC1/Ab-f	Forward	5′-aaaacatatgCTGTTATCAAGAAGCTCGAAGG-3′
SCRC1/Ab-r	Reverse	5′-aaaactcgagtcaTTGCTGGCCCACCTCAACG-3′
BQ2/Ab-f	Forward	5′-aaaacatatgAACTGGATTCGGACACCTATAC-3′
BQ2/Ab-r	Reverse	5′-aaaactctgagtcaTTCATCGGCCAGATAAAAGTGC-3′

* Primers Ag1–Ag8 were for cloning partial gene regions of SCRC1 or SCRBQ2; Primers designated dsRNA- were used in synthesis of DNA templates for amplification of dsRNAs for RNAi experiments. Primers designated qPCR were used for RT-RT-qPCR. Primers designated Ab were used for amplifying and as SCR region for antiserum production.

## Data Availability

Data are contained within the article and Supplementary Materials.

## Supplementary Materials

The following supporting information can be downloaded at the following link: https://www.mdpi.com/article/10.3390/toxins16080369/s1, Figure S1: SCRC1 sequence and protein features; Figure S2: SCRBQ2 sequence and protein features; Figure S3: CLUSTAL O multiple sequence alignment of Ag55 SCRC1 (GH) and SCRC1 homologues from *A. arabiensis*, *A. coluzzii* and *A. gambiae*; and Figure S4: CLUSTAL O multiple sequence alignment of Ag55 SCRBQ2 (GH) and SCRBQ2 homologues from *A. arabiensis*, *A. coluzzii* and *A. gambiae*; Figure S5: CLUSTAL 2.1 multiple sequence alignment of Ag55 SCRC1 (GH) with Aedes aegypti homologues.
