# Supplementary material for: Scavenger Receptor C1 Mediates Toxicity of Binary Toxin from Lysinibacillus sphaericus to Ag55 Cells
Source: Toxins (Basel). 2024 Aug 21;16(8):369. doi: 10.3390/toxins16080369 (PMC11359365; doi:10.3390/toxins16080369)
Supplement: Supplementary file 1 [file toxins-16-00369-s001.zip › toxins-3111987-supplementary.pdf]

toxins

MDPI

Supplementary Material: Scavenger Receptor C1 Mediates Toxicity of Binary Toxin from *Lysinibacillus sphaericus* to Ag55 Cells

SCR-C1 sequence

```
   1 - ATGCTGTTATCAAGAAGCTCGAAGGGAGCAACCCTGCTTGGGGGGTTGTTGTGGATCGTTTTCTTCGCCAATTTGGTCGCTGGCCAATTAACCCTTTACGGCTCGAGAGCGCCCTCCATG - 120
   1 - M  L  L  S  R  S  S  K  G  A  T  L  L  G  G  L  L  W  I  V  F  F  A  N  L  V  A  G  Q  L  T  L  Y  G  S  R  A  P  S  M  - 40

 121 - CGTCGTCCGCTCACTAGACAGTTGGTGGGGCAATGCCCTGCGCCGTACTTCCCGAACGGGGAGGCGAAAATTCGAAACCGTGGCCGAATGATGCGCTTCGACTGTGCGTACGGGTTTAAG - 240
  41 - R  R  P  L  T  R  Q  L  V  G  Q  C  P  A  P  Y  F  P  N  G  E  A  K  I  R  N  R  G  R  M  M  R  F  D  C  A  Y  G  F  K  - 80

 241 - CTGGTCGGCAATCGGTACTCCAACTGCCAGAACGGTCGCTGGGACACATCGATACCGGTTTGTGTGAAATCCGGATGCAGCCTGCTCGCCCCAATAGAGTCCGGCCACGTGCTGTACGAG - 360
  81 - L  V  G  N  R  Y  S  N  C  Q  N  G  R  W  D  T  S  I  P  V  C  V  K  S  G  C  S  L  L  A  P  I  E  S  G  H  V  L  Y  E  - 120

 361 - ATGAACAAAGCGTCCGCCTTTCTGTCCTGCTTTGAGGGGACCGAGCTGGCCGGCTCGAGAAACGCCTACTGCAACGGGACGCACTGGGATCGACCGCTCGGTATCTGCCGGCGTACGGGA - 480
 121 - M  N  K  A  S  A  F  L  S  C  F  E  G  T  E  L  A  G  S  R  N  A  Y  C  N  G  T  H  W  D  R  P  L  G  I  C  R  R  T  G  - 160

 481 - CAGGCGACACCGACGGCCTGCGACTTCGAGACCGAGTCGCTGTGCGGCTGGTCCAACGATGCGCTGCACGATTTCGACTGGAAGCGCAGCGACGGTACGCTGAACCCGCGCGCACTCCGA - 600
 161 - Q  A  T  P  T  A  C  D  F  E  T  E  S  L  C  G  W  S  N  D  A  L  H  D  F  D  W  K  R  S  D  G  T  L  N  P  R  A  L  R  - 200

 601 - ACCGGGCCCAAGTACGACCACACCACGATGCAGCCGAAGGCGGGCCACTTCATGATAGTGGATTCCGGCGAGCAGCTAACGAACGATACGGCCCGCTTCATATCGCCGCTGTTCGAGCCG - 720
 201 - T  G  P  K  Y  D  H  T  T  M  Q  P  K  A  G  H  F  M  I  V  D  S  G  E  Q  L  T  N  D  T  A  R  F  I  S  P  L  F  E  P  - 240

 721 - GAGCTGAGCGTCGGTGCGTGCTTCCAGTTTTACTATCACATGTACGGGGAGTCGGTCGGCACGCTGAAGGTGTTTGTGAAGCCGATGAGCGCCGACCTGTACGATCTGCAGCCGGTGTTT - 840
 241 - E  L  S  V  G  A  C  F  Q  F  Y  Y  H  M  Y  G  E  S  V  G  T  L  K  V  F  V  K  P  M  S  A  D  L  Y  D  L  Q  P  V  F  - 280

 841 - GTGCAGCGGGGCAATCAGAAGAACGTTTGGCACGAGGGTCACGTTGAGGTGGGCCAGCAAGCGGAGCGCTTTCAGGTGGTGATCGAGGCGAGCCTGGGCATGCGCTACAAGAGCGACATT - 960
 281 - V  Q  R  G  N  Q  K  N  V  W  H  E  G  H  V  E  V  G  Q  Q  A  E  R  F  Q  V  V  I  E  A  S  L  G  M  R  Y  K  S  D  I  - 320

 961 - GCGATCGACGATGTGAGCTTGCTGCAGGGCGACAGTTGCCGGGTGGCGGTCGAGGATGGCGAGGAGCCACCACCGGCCGAGGTGGAGAACGTGCCGGTGAAGATAGAGTCGTGCGAAAAC - 1080
 321 - A  I  D  D  V  S  L  L  Q  G  D  S  C  R  V  V  E  D  G  E  E  P  P  P  A  E  V  E  N  V  P  V  K  I  E  S  C  E  N  - 360

1081 - CGGTGCGGAAGCAGTATGGCCGCCGTGCTGAACGCGAACGATACGATTGTGCACTGCGACTGCCACGAGGACTGTGTGACGAGTGAGACCTGCTGTCCGGATTACCGGGAACGGTGCGTT - 1200
 361 - R  C  G  S  S  M  A  A  V  L  N  A  N  D  T  I  V  H  C  D  C  H  E  D  C  V  T  S  E  T  C  C  P  D  Y  R  E  R  C  V  - 400

1201 - TTTCAGGTAGCGGTTGGAGGGAGTAACGTAAGTACGACCACACCCGTTCCTGTACCGTCGACGTCTACCACGGTTAGGACAACTACGGCAAAAGCTGCTGCCACTACGTCTACGCGGCCG - 1320
 401 - F  Q  V  A  V  G  G  S  N  V  S  T  T  T  P  V  P  V  P  S  T  S  T  T  V  R  T  T  T  A  K  A  A  A  T  T  S  T  R  P  - 440

1321 - GTGACAACGACTTCCACAACGGTCAGTAGCACTACTACCACCACTACCACCACCACCACCACAACCACTGCAAAGCCAACAACGTCAAGCGCTACTACGACCAGGAGTACCACTACAAAA - 1440
 441 - V  T  T  T  S  T  T  V  S  S  T  T  T  T  T  T  T  T  T  T  T  T  T  A  K  P  T  T  S  S  A  T  T  T  R  S  T  T  T  K  - 480

1441 - CCATCCACGACGTCCACTGTCTCCACGACAACCACGAAGAAGTACAATCTACGCCCAAGGCCTAGTCTCGTTCCGCTTACCAATCGACCACAGCTGCCACCAACAACCACACCAGCTGCA - 1560
 481 - P  S  T  T  S  T  V  S  T  T  T  T  K  K  Y  N  L  R  P  R  P  S  L  V  P  L  T  N  R  P  Q  L  P  P  T  T  T  P  A  A  - 520

1561 - ACGACTCGCAAAACAACTACGCCAACGACCACAGCCACCTCCACAGCGGTGACAACGACCATCCCCACCACCACTCAATCCGACCTGGTGGCGGTTGAACAGTCCGTGCCATCAACAACC - 1680
 521 - T  T  R  K  T  T  T  P  T  T  T  A  T  S  T  A  V  T  T  T  I  P  T  T  T  Q  S  D  L  V  A  V  E  Q  S  V  P  S  T  T  - 560

1681 - GAGGAGGTGATAATGCCCGGCCTGGCCATGGCCCCACTGCAACCGGTAGAAAAACTCCACTCCACTCCGAGTCTGATGAAGTTCTTCGTGTACGCCATATCGACCGTAGTGCTGTTTGCG - 1800
 561 - E  E  V  I  M  P  G  L  A  M  A  P  L  Q  P  V  E  K  L  H  S  T  P  S  L  M  K  F  F  V  Y  A  I  S  T  V  V  L  F  A  - 600

1801 - TGCATTTTAAGCGCTGCGTACCTTTACGCACGACGGTCGCGTTCCAGCGTGCTGGCAAGGTTGAAGGAAAAATCGCAAAAGAGTGGCTTCGAGGACATCCGGTTTCTGGCTGGCGATGAA - 1920
 601 - C  I  L  S  A  A  Y  L  Y  A  R  R  S  R  S  S  V  L  A  R  L  K  E  K  S  Q  K  S  G  F  E  D  I  R  F  L  A  G  D  E  - 640

1921 - GACTTAGACTTTAACATTACGCACGGGCGCGATGTGGAGGAGGGAGAGGAGGTGCTGGAAAAGGAAGGCAAAGGGGAGGCAAAAGCAAAACAGGAAACCAAACCAAGCAAGAAGGACGCA - 2040
 641 - D  L  D  F  N  I  T  H  G  R  D  V  E  E  G  E  E  V  L  E  K  E  G  K  G  E  A  K  A  K  Q  E  T  K  P  S  K  K  D  A  - 680

2041 - AAAGCGGACATC - 2052
 681 - K  A  D  I   - 720
```

Figure S1. Sequence of scavenger receptor C1 (SCRC1): signal leading peptide (bold) and transmembrane domain were underlined with red; CCP (complement control protein) domain (aa 49-103) was underlined with blue and MAM domain (aa 162-335) underlined with orange. Black underline denotes region expressed for preparing antisera.

SCR-BQ2 sequence

```
   1 - tggcgtaggcaaagtggtggaatcattgtgtagcgggtgaaaataaagcacactggatgagctgataagagggacaacagtgcgcgtgacgacaccaaaacctaccgccacaaggacagc - 120

 121 - ggccataaaataccaaagcaatatagaggcgttataacgcaatccacccaaaacaaaatattgccctcagctgcaaactacaATGTGTTCACCATGTACAGACTTTCAGAAAAAGTTTAT - 240
   1 -                                                                                   M  C  S  P  C  T  D  F  Q  K  K  F  I  - 13
 241 - ATCACTAGGATGTTCGTCCTTTCTAATCTTGCTAGCGATTACACTAGGCGTGCTTTGGCCATCCCTGTCCGACCAGGTGCTGCACAATAAACTGGTTATTAAAAATGGATCGTCGAACTA - 360
  14 -  S  L  G  C  S  S  F  L  I  L  L  A  I  T  L  G  V  L  W  P  S  L  S  D  Q  V  L  H  N  K  L  V  I  K  N  G  S  S  N  Y  - 53
 361 - TGACAACTGGATTCGGACACCTATACCCATGTATCTGGAGGTATATTTCTTTAACTGGACTAATCCGGATGACGTAAAGACGAAGAACGGTACAAAGCCACACTTTGTGGAGATGGGACC - 480
  54 -  D  N  W  I  R  T  P  I  P  M  Y  L  E  V  Y  F  F  N  W  T  N  P  D  D  V  K  T  K  N  G  T  K  P  H  F  V  E  M  G  P  - 93
 481 - GTACACGTTTTCCGAGGTTCACGAACGTGTCAACCTAGTCTGGAACGCTAACAGTACGGTAACATATGAGCAGCGCAGGACGTGGCATTTTGTACCGGAGCTATCGAAAGGCACACTGGA - 600
  94 -  Y  T  F  S  E  V  H  E  R  V  N  L  V  W  N  A  N  S  T  V  T  Y  E  Q  R  R  T  W  H  F  V  P  E  L  S  K  G  T  L  D  - 133
 601 - TGATCAAGTTACAAATTTGAACGTTATCACCTTGAATGCTGCACATTTTTTACGCAATACATATCCCCTGCTTAGACCACTAATAAATATATTCCTTAAAACGGATGGTTCGTTGCTGTG - 720
 134 -  D  Q  V  T  N  L  N  V  I  T  L  N  A  A  H  F  L  R  N  T  Y  P  L  L  R  P  L  I  N  I  F  L  K  T  D  G  S  L  L  W  - 173
 721 - GAAAAACAAACCAGTGCGCGAGCTGCTGTTCGAAGGCGTTAAAGATCCACTGCTAGATCTGCTGAAGACTATCAATAGCACATCACTGAACATACCGTTTGATAAGTTTGGCTGGTTTGT - 840
 174 -  K  N  K  P  V  R  E  L  L  F  E  G  V  K  D  P  L  L  D  L  L  K  T  I  N  S  T  S  L  N  I  P  F  D  K  F  G  W  F  V  - 213
 841 - TGGGCGTAATCTGAGCGACACGTTTGACGGCACCTTCACGATGAGAACGGGTGCGGATGGGCTGGAAAGCATGGGCTTCCTAACGCAGTGGAACGGTTCACCCAACACCGGCATGTACCG - 960
 214 -  G  R  N  L  S  D  T  F  D  G  T  F  T  M  R  T  G  A  D  G  L  E  S  M  G  F  L  T  Q  W  N  G  S  P  N  T  G  M  Y  R  - 253
 961 - GGGAAAGTGCGGCGAAGTGTACGGTACGTCCGGCGAGCTTTGGCCAGCCTCTAGCAATGTTCCGGCAAACATTACACTCTTCCCATCGGACATTTGCCGTTCCATTACGCTGCAAGGCAG - 1080
 254 -  G  K  C  G  E  V  Y  G  T  S  G  E  L  W  P  A  S  S  N  V  P  A  N  I  T  L  F  P  S  D  I  C  R  S  I  T  L  Q  G  R  - 293
1081 - GGAACAAGTTTCGCTGTACAACATACAGGGCACGAAGTACGTTGGCGACGATCGTGTTTTCGACAACGGTGTAAAATACCCCGAGGCTAGCTGCTGGTGCAACTCCAATCCCACCCAATG - 1200
 294 -  E  Q  V  S  L  Y  N  I  Q  G  T  K  Y  V  G  D  D  R  V  F  D  N  G  V  K  Y  P  E  A  S  C  W  C  N  S  N  P  T  Q  C  - 333
1201 - CCCTGACCTTAAGCCGGGTGTGTTTAATGCTTCCGCCTGCAAATATGGATCGCCAACGTTCGTATCGTTTCCGCACTTTTATCTGGCCGATGAAAGCTACCAAACTGCTGTAACGGGACT - 1320
 334 -  P  G  L  K  P  G  V  F  N  A  S  A  C  K  Y  G  S  P  T  F  V  S  F  P  H  F  Y  L  A  D  E  S  Y  Q  T  A  V  T  G  L  - 373
1321 - GCGGCCAAATCAAACGGAGCACGAGTTTTACATGGCAATCGAGCCATCGACTGGCATTCCGTTGGACGTTCGTGCCCAGCTGCAGATCAATGAGCACTTGCAGCCGATAAAAGGGTTTAG - 1440
 374 -  R  P  N  Q  T  E  H  E  F  Y  M  A  I  E  P  S  T  G  I  P  L  D  V  R  A  Q  L  Q  I  N  E  H  L  Q  P  I  K  G  F  S  - 413
1441 - TTTCTACGAGCATGTACCGGACGTAATGGTACCGATGATCTGGTTCCGACAACGTGCAACCCTGACGCAAGAGCTGGCAGAACAGGCGAAGCTTGCACTGGCACTACCGTCGCTCGGCAT - 1560
 414 -  F  Y  E  H  V  P  D  V  M  V  P  M  I  W  F  R  Q  R  A  T  L  T  Q  E  L  A  E  Q  A  K  L  A  L  A  L  P  S  L  G  I  - 453
1561 - CTACGTGGCGGTCTTCTTTGGCTCTATCGGTATTATCATGACGTCCGTATTCCTATTCTGCAGTATCAAAAAATGGTCCCAAAGCGCAGAGGTAGTTCCGTACGAAGAGCTACAAAATTA - 1680
 454 -  Y  V  A  V  F  F  G  S  I  G  I  I  M  T  S  V  F  L  F  C  S  I  K  K  W  S  Q  S  A  E  V  V  P  Y  E  E  L  Q  N  *  - 492
1681 - Acgtgttaaaagcatcgacgcaaacaaaaaatataca
```

Figure S2. Sequence of scavenger receptor BQ2: signal leading peptide (bold) and transmembrane domain were underlined with red. The region of SCRBQ2 expressed for preparing antisera is underlined in black.

Figure S3. CLUSTAL O (1.2.4) multiple sequence alignment of Ag55 SCRC1 (GH) and SCRC1 homologues from *A. arabiensis* (XP_040167338.1), *A. coluzzii* (XP_040235850.2), and *A. gambiae* XP_061518211.1

```
SCRC1_GH        -----------MLLSRSSKGATLLGGLLWIVFFANLVAGQLTLYGSRAPSMRRPLTRQLV       49
arabiensis      -----------MLLSRSSKGATLLGGLLWIVFFANLVAGQLTLYGSRAPSMRRPLTRQLV       49
coluzzii        MCAYCSRILPPMLLSRSSKGATLLGGLLWIVFFANLVAGQLTLYGSRAPSMRRPLTRQLV       60
gambiae         -----------MVLSRSLKGATLLGGLLWIVFFANLVAGQLTLYGSRAPSMRRPLTRQLV       49
                           *:**** ******************************************

SCRC1_GH        GQCPAPYFPNGEAKIRNRGRMMRFDCAYGFKLVGNRYSNCQNGRWDTSIPVCVKSGCSLL       109
arabiensis      GQCPAPYFPNGEAKIRNRGRMMRFDCAYGFKLVGNRYSNCQNGRWDTSIPVCVKSGCSLL       109
coluzzii        GQCPAPYFPNGEAKIRNRGRMMRFDCAYGFKLVGNRYSNCQNGRWDTSIPVCVKSGCSLL       120
gambiae         GQCPAPYFPNGEAKIRNRGRMMRFDCAYGFKLVGNRYSNCQNGRWDTSIPVCVKSGCSLL       109
                ************************************************************

SCRC1_GH        APIESGHVLYEMNKASAFLSCFEGTELAGSRNAYCNGTHWDRPLGICRRTGQATPTACDF       169
arabiensis      APIESGHVLYEMNKASAFLSCFEGTELAGSRNAYCNGTHWDRPLGICRRTGQATPTACDF       169
coluzzii        APIESGHVLYEMNKASAFLSCFEGTELAGSRNAYCNGTHWDRPLGVCRRTGQATPTACDF       180
gambiae         APIESGHVLYEMNKASAFLSCFEGTELAGSRNAYCNGTHWDRPLGVCRRTGQATPTACDF       169
                *********************************************:**************

SCRC1_GH        ETESLCGWSNDALHDFDWKRSDGTLNPRALRTGPKYDHTTMQPKAGHFMIVDSGEQLTND       229
arabiensis      ETESLCGWSNDALHDFDWKRSDGTLNPRALRTGPKYDHTTMQPKAGHFMIVDSGEQLTND       229
coluzzii        ETESLCGWSNDALHDFDWKRSDGTLNPRALRTGPKYDHTTMQPKAGHFMIVDSGEQLTND       240
gambiae         ETESLCGWSNDALHDFDWKRSDGTLNPRALRTGPKYDHTTMQPKAGHFMIVDSGEQLTND       229
                ************************************************************

SCRC1_GH        TARFISPLFEPELSVGACFQFYYHMYGESVGTLKVFVKPMSADLYDLQPVFVQRGNQKNV       289
arabiensis      TARFISPLFEPELSVGACFQFYYHMYGESVGTLKVFVKPMSADLYDLQPVFVQRGNQKNV       289
coluzzii        TARFISPLFEPELSVGACFQFYYHMYGESVGTLKVFVKPMSADLYDLQPVFVQRGNQKNV       300
gambiae         TARFISPLFEPELSVGACFQFYYHMYGESVGTLKVFVKPMSADLYDLQPVFVQRGNQKNV       289
                ************************************************************

SCRC1_GH        WHEGHVEVGQQAERFQVVIEASLGMRYKSDIAIDDVSLLQGDSCRVAVEDGEEPPPAEVE       349
arabiensis      WHEGHVEVGQQAERFQVVIEASLGMRYKSDIAIDDVSLLQGDSCRVAVEDGEEPPPAEVE       349
coluzzii        WHEGHVEVGQQAERFQVVIEASLGMRYKSDIAIDDVSLLQGDSCRVAVEDGEEPPPAEVE       360
gambiae         WHEGHVEVGQQAERFQVVIEASLGMRYKSDIAIDDVSLLQGDSCRVAVEDGEEPPPAEVE       349
                ************************************************************

SCRC1_GH        NVPVKIESCENRCGSSMAAVLNANDTIVHCDCHEDCVTSETCCPDYRERCVFQVAVGGSN       409
arabiensis      NVPVKIESCENRCGSSMAAVLNANDTIVHCDCHEDCVTSETCCPDYRERCVFQVAVGGSN       409
coluzzii        NVPVKIESCENRCGSSMAAVLSANDTIVHCDCHEDCVTSETCCPDYRERCVFQVAVGGSN       420
```

```
gambiae         NVPVKIESCENRCGSSMAAVLNANDTIVHCDCHEDCVTSETCCPDYRERCVFQVAVGGSN       409
                *********************.**************************************

SCRC1_GH        VSTTTPVPVPSTSTTVRTTTAKAAATTSTRPVTTTSTTVSSTTTTTT-TTTTTTTAKPTT       468
arabiensis      VSTTTPVPVPSTSTTVRTTTAKAAATTSTRPVTTTSTTVSSTTTTTT-TTTTTTTAKPTT       468
coluzzii        VSTTTPVPVPSTSTTVRTTTAKAAATTSTRPVTTTSTTVSSTTTTTTTTTTTTTTAKPTT       480
gambiae         VSTTTPVPVPSTSTTVRTTTAKAAATTSTRPVTTTSTTVSSTTTT----TTTTTTAKPTT       465
                *********************************************    ***********

SCRC1_GH        SSATTTRSTTTKPSTTSTVSTTTTKKYNLRPRPSLVPLTNRPQLPPTTTPAATTRKTTTP       528
arabiensis      SSATTTRSTTTKPSTTSTVSTTTTKKYNLRPRPSLVPLTNRPQLPPTTTPAATTRKTTTP       528
coluzzii        SSATTTRSTTTKPTTTSTVSTTTTKKYNLRPRPSLVPLTNRPQLPPTTTPAATTRKTTTP       540
gambiae         SSATTTRSTTTKPTTTSTVSTTTTKKYNLRPRPSLVPLTNRPQLPPTTTPAATTRKTTTP       525
                *************:**********************************************

SCRC1_GH        TTTATSTAVTTTIPTTTQSDLVAVEQSVPSTTEEVIMPGLAMAPLQPVEKLHSTPSLMKF       588
arabiensis      TTTATSTTVTTTIPTTTQSDLVAVEQSVPSTTEEVIMPGLAMAPLQPVEKLHSTPSLMKF       588
coluzzii        TTTATSTTVTTTIPTTTQSDLVAVEQSVPSTTEEVIMPGLAMAPLQPVEKLHSAPSLMKF       600
gambiae         TTTATSTTVTTTIPTTTQSDLVAVEQSVPSTTEEVIMPGLAMAPLQPVEKLHSAPSLMKF       585
                *******:*********************************************:******

SCRC1_GH        FVYAISTVVLFACILSAAYLYARRSRSSVLARLKEKSQKSGFEDIRFLAGDEDLDFNITH       648
arabiensis      FVYAISTVVLFACILSAAYLYARRSRSSVLARLKEKSQKSGFEDIRFLAGDEDLDFNITH       648
coluzzii        FVYAISTVVLFACILSAAYLYARRSRSSVLARLKEKSQKSGFEDIRFLAGDEDLDFNISH       660
gambiae         FVYAISTVVLFACILTAAYLYARRSRSSVLARLKEKSQKSGFEDIRFLAGDEDLDFNISH       645
                ***************:******************************************:*

SCRC1_GH        GRDVEEGEEVLEKEGKGEAKAKQETKPSKKDAKADIVRMAKQEDKKNLATHGRGEEDSNT       684
arabiensis      GRDVEEGEEVLEKEGKGEAKAKQETKPSKKDAKADIVRMAKQEDKKNLATHGRGEEDSNT       708
coluzzii        GRDVEEGEEVLEKKGNGEAKAKQETKSSKKDAKADIVRMAKQEDKKNLATHGRGEEDSNT       720
gambiae         GRDVEEGEEVLEKKGNGEAKAKQETKPSKKDAKADIVRMAKQEDKKNLATHGRGEEDSNT       705
                *************:*:********** *********************************

SCRC1_GH        DSDASTDDDGAGGGGAKRTHGKPNKKAYQKYVKHHDEDMASSLL     752
arabiensis      DSDASTDDDGAGGGGAKRTHGKPNKKAYQKYVKHHDEDMASSLL     752
coluzzii        DSDASTDDDGAGGGGAKRTHGKPNKKAYQKYVKHHDEDMASSLL     764
gambiae         DSDASTDDDGAGGGGAKRTHGKPNKKAYQKYVKHHDEDMASSLL     749
                                   ********************************************
```

Figure S4. CLUSTAL O (1.2.4) multiple sequence alignment of Ag55 SCRBQ2 (GH) and SCRBQ2 homologues from *A. arabiensis* (XP_040167338.1), *A. coluzzii* (XP_040234709.2), and *A. gambiae* (XP_061510019.1)

```
BQ2-GH          MCSPCTDFQKKFISLGCSSFLILLAITLGVLWPSLSEQVLHNKLVIKNGSSNYDNWIRTP       60
gambiae         MCSPCTDFQKKFISLGCSSFLILLAITLGVLWPSLSEQVLHNKLVIKNGSSNYDNWIRTP       60
arabiensis      MCSPCTDFQKKFISLGCSSFLILLAITLGVLWPSLSEQVLHNKLVIKNGSSNYDNWIRTP       60
coluzzii        MCSPCTDFQKKFISLGCSSFLILLAITLGVLWPSLSEQVLHNKLVIKNGSSNYDNWIRTP       60
                ************************************************************

BQ2-GH          IPMYLEVYFFNWTNPDDVKTKNGTKPHFVEMGPYTFSEVHERVNLVWNANSTVTYEQRRT       120
gambiae         IPMYLEVYFFNWTNPDDVKTKNGTKPHFVEMGPYTFSEVHERVNLVWNANSTVTYEQRRT       120
arabiensis      IPMYLEVYFFNWTNPDDVKTKNGTKPHFVEMGPYTFSEVHERVNLVWNANSTVTYEQRRT       120
coluzzii        IPMYLEVYFFNWTNPDEVKTKNGTKPHFVEMGPYTFSEVHERVNLVWNANSTVTYEQRRT       120
                ****************:*******************************************

BQ2-GH          WHFVPELSKGTLDDQVTNLNVITLNAAHFLRNTYPLLRPLINIFLKTDGSLLWKNKPVR-       180
gambiae         WHFVPELSKGTLDDQVTNLNVITLNAAHFLRNTYPLLRPLINIFLKTDGSLLWKNKPVRE       180
arabiensis      WHFVPELSKGTLDDQVTNLNVITLNAAHFLRNTYPLLRPLINIFLKTDGSLLWKNKPVRE       180
coluzzii        WHFVPELSKGTLDDQVTNLNVITLNAAHFLRNTYPLLRPLINIFLKTDGSLLWKNKPVRE       180
                ***********************************************************

BQ2-GH          LLFEGVKDPLLDLLKTINSTSLNIPFDKFGWFVGRNLSDTFDGTFTMRTGADGLESMGFL       240
gambiae         LLFEGVKDPLLDLLKTINSTSLNIPFDKFGWFVGRNLSDTFDGTFTMRTGADRLESMGFL       240
arabiensis      LLFEGVKDPLLDLLKTINSTSLNIPFDKFGWFVGRNLSDTFDGTFTMRTGADGLESMGFL       240
coluzzii        LLFEGVKDPLLDLLKTINSTSLNIPFDKFGWFVGRNLSDTFDGTFTMRTGADGLESMGFL       240
                **************************************************** *******

BQ2-GH          TQWNGSPNTGMYRGKCGEVYGTSGELWPASSNVPANITLFPSDICRSITLQGREQVSLYN       300
gambiae         TQWNGSPNTGMYRGKCGEVYGTSGELWPASSNVPANITLFPSDICRSITLQGREQVSLYN       300
arabiensis      TQWNGSPNTGMYRGKCGEVYGTSGELWPASSNVPANITLFPSDICRSITLQGREQVSLYN       300
coluzzii        TQWNGSPNTGMYRGKCGEVYGTSGELWPASSNVPANITLFPSDICRSITLQGREQVSLYN       300
                ************************************************************

BQ2-GH          IQGTKYVGDDRVFDNGVKYPEASCWCNSNPTQCPGLKPGVFNASACKYGSPTFVSFPHFY       360
gambiae         IQGTKYVGDDRVFDNGVKYPEASCWCNSNPTQCPDLKPGVFNASACKYGSPTFVSFPHFY       360
arabiensis      IQGTKYVGDDRVFDNGVKYPEASCWCNSNPTQCPDLKPGVFNASACKYGSPTFVSFPHFY       360
coluzzii        IQGTKYVGDDRVFDNGVKYPEASCWCNSNPTQCPDLKPGVFNASACKYGSPTFVSFPHFY       360
                **********************************.*************************

BQ2-GH          LADESYQTAVTGLRPNQTEHEFYMAIEPSTGIPLDVRAQLQINEHLQPIKGFSFYEHVPD       420
gambiae         LADESYQTAVTGLRPNQTEHEFYMAIEPSTGIPLDVRAQLQINEHLQPIKGFSFYEHVPD       420
arabiensis      LADESYQTAVTGLRPNQTEHEFYMAIEPSTGIPLDVRAQLQINEHLQPIKGFSFYEHVPD       420
```

```
coluzzii      LADESYQTAVTGLRPNQTEHEFYMAIEPSTGIPLDVRAQLQINEHLQPIKGFSFYEHVPD   420
              ************************************************************

BQ2-GH        VMVPMIWFRQRATLTQELAEQAKLALALPSLGIYVAVFFGSIGIIMTSVFLFCSIKKWSQ   480
gambiae       VMVPMIWFRQRATLTQELAEQAKLALALPSLGIYVAVFFGSIGIIMTSVFLFCSIKKWSQ   480
arabiensis    VMVPMIWFRQRATLTQELAEQAKLALALPSLGIYVAVFFGSIGIIMTSVFLFCSIKKWSQ   480
coluzzii      VMVPMIWFRQRATLTQELAEQAKLALALPSLGIYVAVFFGSIGIIMTSVFLFCSIKKWSQ   480
              ************************************************************

BQ2-GH        SAEVVPYEELQN   492
gambiae       SAEVVPYEELQN   492
arabiensis    SAEVVPYEELQN   492
coluzzii      SAEVVPYEELQN   492
              ************
```

Figure S5. CLUSTAL 2.1 multiple sequence alignment of Ag55 SCRC1 (GH) with *Aedes aegypti* homologues. A BLAST search with Ag55 SCRC1 protein identified XP_021710624. A second scavenger receptor called AeSC-C (AAEL006361) in *A. aegypti* is reported by Xiao et al. (2014. Plos Pathogens, 10: e1004027) in their in their study of flavivirus and the role of inducing antimicrobial peptides and control of flavivirus infections.

```
AgSCRC1(GH)        --MLLSRSSKGATLLGGLLWIVFFANLVAGQLTLYGSRAPSMRRPLTRQLVGQCPAPYFP
XP_021710624       MFAHLSEVSILAVALWLALEISSTTGLVQYRLTPLRTGGRPMKQPQLKSGP-ECPTPYFP
AeSC-C             ----MNIDRLSQTFIFVVLALVVDISAYDGRYSPVLRNSYSN----------ICPKPEFQ
                       :.      . :   * :    .    : :     . .            ** * *

AgSCRC1            NGEAKIRNRGRMMRFDCAYGFKLVGNRYSNCQNGRWDTSIPVCVKSGCSLLAPIESGHVL
XP_021710624       NGVAKPRQRNKMIRFTCASGFTLVGNMYSMCEKGRWDTPIPICIRSGCPDLELVANGQIS
AeSC-C             NGFIRMRLRGKYLRFECYADFTLVGLPLLLCKNGRWNSQVPICVKPGCSDLSAVDNGFIF
                   **  : * *.: :** *  .*.***     *::***:: :*:*::.**. *  : .* :

AgSCRC1            YEMNKASAFLSCFEGTELAGSRNAYCNGTHWDRPLGICRRTGQATPTACDFETESLCGWS
XP_021710624       YEYGQAAAMLFCSSGYQVAGASKAYCNGTHWDRPLGNCRETGIGVETSCDFEAPDLCGWM
AeSC-C             YNNMKAAATLFCDSGYKLAGSMYSYCNGTAWDRSLGSCRRTNWTVAVACDFEVSDLCGWE
                   *:  :*:* * * .* ::**:  :***** ***.** **.*.  . .:****. .****

AgSCRC1            NDALHDFDWKRSDGTLNPRALRTGPKYDHTTMQPKAGHFMIVDSGEQLTNDTARFISPLF
XP_021710624       NEATHDFDWKRSDGIVHPKALKTGPKFDHTTGSALAGHFMMVDSVEQFTNETARLLSPMY
AeSC-C             SEATHRLDWERSSGDASRRARKTGPTHDHTTGEPLNGHFMMVDSSQANMNETARFISPIY
                   .:* * :**:**.*    :* :***..****  ..  ****:*** :   *:***::**::

AgSCRC1            EPELSVGACFQFYYHMYGESVGTLKVFVKPMSADLYDLQP---VFVQRGNQKNVWHEGHV
XP_021710624       PANYSTNACFSFYYHMYGDGVGTLSVYVRPASQQLDSYSSKDAIFSLKGNQRNVWNEGYF
AeSC-C             STNYSKNACFRLFYHMFGD-AGTLAIYVKPLSVNLREMAKQSAPFSVSGNQGNAWIEGCL
                    .: * .*** ::***:*: .*** ::*:* * :* .       *   *** *.* ** .

AgSCRC1            EVGQQAERFQVVIEASLGMRYKSDIAIDDVSLLQGDSCRVAVEDGEE-------PPPAEV
XP_021710624       DLKQFSEEFQIVIEASLGMKAKSDIAIDDVSLLYGEDCRPMAESEDEEITPEVLPPDVPS
AeSC-C             DLEQQNESFQIVIQASLGMRYKSDVAIDDVQLFGGCNCNSNGTSEEP---------VTES
                   :: *  * **:**:*****: ***:*****.*: * .*.    . :           .

AgSCRC1            ENVPVKIESCENRCGSSMAAVLNANDT---IVHCDCHEDCVTSETCCPDYRERCVFQVAV
XP_021710624       DDNIFKLGSCENRCGVNVSSVVTIPDTSVNFVQCDCFEGCVDSKTCCPDYAERCVFNETA
AeSC-C             DDDMFQIDSCVGRCGQDVSDSSDVGERS--MIHCGCNNDCNEDNTCCPDYVELCVPDE--
                   ::  .:: ** .*** .::      :    :::*.* :.*  .:****** * ** :

AgSCRC1            GGSNVSTTTPVPVPSTSTTVRTTTAKAAATTSTRPVTTTSTTVSSTTTTTTTTTTT--TT
XP_021710624       FAASTISTSTKILPSTTETTRPTTTPTTSTTTTTIRTTQPTTKPTTTPTKPTTTSTRPTT
```

```
AeSC-C          ------------------------------------------------------------


AgSCRC1         AKPTTSSATTTRSTTTKPSTTSTVSTTTTKKYNLRPRPSLVPLTNRPQLPPTTTPAATTR
XP_021710624    PRSTSTTAVQTSSSTTIKTTLKPKVPTTTTTKSYNLRPRNITTTSSTTTKPMLLPMVRTR
AeSC-C          ----STAADGYEDQFKFPVTPKPIGSNSCKAMEFMG------------------------
                    :::*    .  .   * ..  ..: .  .

AgSCRC1         KTTTPTTTATSTTVTTTIPTTTQSDLVAVEQSVPSTTEEVIMPGLAMAPLQPVEKLHSTP
XP_021710624    RPTTSTTTTTST------PTTEEVVLKLVARFGDVSSEEFPAS--------VLEYGDNKP
AeSC-C          ------------------------------------------------------------


AgSCRC1         SLMKFFVYAISTVVLFACILSAAYLYARR-SRSSVLARLKEKSQKS------GFEDIRFL
XP_021710624    NLARVFLFSGIGVVSFGCVILLIVLHIRRNSGENVLNRLKQKSMKSKLGSNEGFEDVRFL
AeSC-C          ----IFLIILSIIMAMICFSQTSRSQISSMFRKIRPNRVDDAFGMA--------EDVQYL
                    .*:     :: : *.             .    *:.:    :        **:::*

AgSCRC1         AGDEDLDFNITHGRDVEEGEEVLEKEGKGEAKAKQETKPSKKDAKADIVRMAKQEDKKNL
XP_021710624    AADEHLDFSLPD-------DEVEEAVEGGDGNNDAEQKPSKKGKK---------------
AeSC-C          AVN-----------------DTISNFSGDNGSVASMRLP---------------------
                * :                 :. .    .:..      *

AgSCRC1         ATHGRGEEDSNTDSDASTDDDGAGGGAKRTHGKPNKKAYQKYVKHHDEDMASSLL
XP_021710624    -------------------------------------------------------
AeSC-C          -------------------------------------------------------
```
